# Care for the cerebrovascular accident survivors: experiences of family caregivers

**DOI:** 10.1186/s12904-024-01468-6

**Published:** 2024-06-01

**Authors:** Fortune Selasi Atsu, Nkosi Nkosi Botha, Cynthia Esinam Segbedzi, Mary Aku Ogum, Daniel Apaak, Ivy Selorm Tsedze, Lucy Adjanor Akoto, Edward Wilson Ansah

**Affiliations:** 1 Department of Health, Physical Education and Recreation (HPER), University of Cape Coast, Cape Coast, Ghana; 2 Department of Adult Health, School of Nursing and Midwifery, University of Cape Coast, Cape Coast, Ghana; 3 E. P. College of Education, Amedzofe, Ghana; 4 Air Force Medical Centre, Air Force Base, Takoradi, Ghana

**Keywords:** Stroke survivors, Family caregivers, Cerebrovascular accident risk, Sustainable development goals, Loss of income, Coping strategies

## Abstract

**Background:**

The role of family caregivers in the management of cerebrovascular accident survivors is invaluable. So far, there is a strong evidence affirming the effectiveness of family support for cerebrovascular accident survivors. Meanwhile, caring for cerebrovascular survivors can be labour and time intensive and pretty stressful for caregivers. The purpose of the study was to examine the lived experiences of family caregivers of cerebrovascular accident survivors in the Ho Municipality in the Volta Region of Ghana. This aims are to establish their caregivers’ knowledge, preparedness, and impact of caregiving on the caregiver, and coping strategies caregivers adopted.

**Methods:**

Using a four-item (with 14 prompts) interview guide and descriptive phenomenological approach, we gathered and analysed data from 37 family caregivers in the Ho Municipality of Ghana.

**Results:**

We found that caregivers had limited knowledge about cerebrovascular disease-risk factors and were ill-prepared for their caregiving roles. Additionally, we found limited knowledge about coping strategies among the caregivers. We further report that some caregivers lost close relationships, and their jobs because of the caregiving, they also used fasting and prayer as coping strategies.

**Conclusions:**

Caregivers suffered broken relationship, loss their jobs and incomes due to their caregiving roles. Moreover, some engaged in fasting and prayers, and alcohol use to cope with the stress associated with caring for the cerebrovascular accident survivors. We discussed the potential implications of our findings on the realisation of the Sustainable Development Goal 3.4. The aim of this goal is to reduce by 75% premature deaths due to cerebrovascular and other non-communicable diseases by 2030.

**Supplementary Information:**

The online version contains supplementary material available at 10.1186/s12904-024-01468-6.

## Introduction

Globally, cerebrovascular accident remains the most predominant non-communicable diseases, implicated in the death of millions individuals, which over 70% are found in the developing countries [1–4]. As a non-communicable disease, cerebrovascular accident is essentially anomalies relating to the heart and associated blood vessels, including coronary heart disease and other vascular anomalies [3–5]. Cerebrovascular accident is a medical emergency condition involving a disruption in blood flow to a part or parts of the brain, depriving the brain from the needed oxygen and nutrients resulting in a functional abnormality of the central nervous system [4, 5]. This deprivation triggers a lasting brain damage, long-term disability, like stroke, heart attack or even death [2, 3]. So far, the world is experiencing a surge in the cerebrovascular accident burden, with developing countries disproportionately impacted [6, 7]. Meanwhile, the Sustainable Development Goals 3.4 aims at reducing by 75% premature deaths due to cerebrovascular and other non-communicable diseases by 2030 [5, 7]. Therefore, family caregivers who provide essential care to cerebrovascular accident survivors have become important actors in our collective quest to reducing mortality due to the condition [8–16]. For the purpose of this study, family caregivers are family members or close friends who provide supportive care to cerebrovascular accident survivors, mainly at home [12–15, 17, 18].

The role of family caregivers in managing cerebrovascular accident survivors is invaluable [8, 10, 11, 13, 15, 16, 19, 20]. There is a growing global recognition of how family care is complementing medical interventions in improving the health and quality of life of cerebrovascular accident survivors [12, 17, 18, 21, 22]. So far, there is a significant agreement across literature [8, 9, 18, 23] affirming the efficacy of family support and care in improving the health quality of life of cerebrovascular accident survivors. Meanwhile, caring for cerebrovascular accident survivors can be labour and time intensive and pretty stressful, because the caregiver may have to contend with complex and sometimes unfamiliar instructions from the medical staff [11, 18, 19, 21]. Moreover, evidence suggests that effective family care and support require time, knowledge, skills, patience, and resources, which are normally very limited or lacking [18, 19]. Considering how intricate and burdensome the caregiver role can be, preparing family caregivers for the task ahead is fundamental [8, 9]. Thus, family caregivers are expected to be knowledgeable in cerebrovascular accident-risk factors and coping strategies to enable them provide effective support to survivors of cerebrovascular accident [15, 16, 18, 21]. Unfortunately, this is not the case in most parts of the world [19, 21]. Meanwhile, there is growing evidence of family caregivers reporting multipsychological conditions like sadness, distress, fear, confusion, and anxiety [9, 22, 47]. Additionally, such caregivers are found to be presenting with cerebrovascular accident-risk factors due to their caregiving roles [13–15, 19–21].

In Africa, family caregiving is a key feature in the management of cerebrovascular accident survivors [24, 25]. Unfortunately, global estimates show a worrying increase in cerebrovascular accident burden, with over 85% of such conditions occurring in the developing and least developed world [6, 7]. Meanwhile, worsening socio-economic factors, socio-cultural factors that define health and disease, disintegration of the extended family tradition, etcetera, are frustrating the work of family caregivers in Africa [15, 19, 21]. Moreover, family caregivers in Africa report low knowledge about the cerebrovascular accident-risk factors and are ill-prepared to provide effective care to cerebrovascular accident survivors [15, 19, 21]. Given the desire to see early recovery, most caregivers fail to pay attention to their own health and quality of life [15, 19, 21]. Meanwhile, stressors associated with caregiving could be insidious, yet may be debilitating to the health and well-being of the caregiver [15, 19, 21].

In Ghana, mortality and morbidity from cerebrovascular accidents are increasing in both urban and rural populations with reported higher incidences [15, 26–33]. Meanwhile, family caregivers have almost become a permanent feature in the management of cerebrovascular accident survivors in Ghana [15, 19, 21]. While evidence abound regarding cerebrovascular accident survivors, research on caregivers is very scanty [15, 19, 21]. For instance, how prepared are family caregivers of cerebrovascular accident survivors in Ghana through training to produce the needed knowledge? What specific barriers do family caregivers of cerebrovascular accident survivors in Ghana face? What are the impacts of caring for cerebrovascular accident survivors on the family caregivers in Ghana? And, what coping strategies are adopted by family caregivers of cerebrovascular accident survivors in Ghana? These questions and more underscore the existing gaps in the literature, and define the scope of the current study. Therefore, this study examined the lived experiences of family caregivers of cerebrovascular accident survivors in the Ho Municipality in the Volta Region of Ghana, to establish their knowledge, preparedness, and impact of their roles, and coping strategies adopted during the caregiving.

## Materials and methods

### Study design

This study adopted descriptive phenomenological approach (DPA) [34, 35] to explore the experiences of family caregivers of cerebrovascular accident survivors in the Ho Municipality of Ghana. This design offers opportunity for probing the rich lived experiences of family caregivers of cerebrovascular accident survivors [37, 38]. Contrary to other designs, the approach is a participant-centred that acknowledges the study context [39, 40]. Moreover, DPA uses techniques such as bracketing, horizontalisation, clustering, and textualisation in upholding the subjective views of participants [34, 36, 40]. However, given that DPA is context specific and dwells on the subjective views of the participants, it is difficult to generalise the findings [38, 40].

### Study participants

In all, 44 family caregivers of cerebrovascular accident survivors in the Ho Municipality were contacted, but seven declined to participate in the study. Therefore, 37 family caregivers (8 males and 29 females) were recruited and participated in this study. According to a source at the Ho Teaching Hospital Cardio Centre, there could be around 50 cases of cerebrovascular accident survivors within the Ho Municipality. On record, the Cardio Centre had contact details of only 13 cerebrovascular accident survivors who were living within the municipality. Clearly, there were cerebrovascular accident survivors within the Municipality who were off the records of the centre. Therefore, we used the contact details provided by the Centre to contact the caregivers within the Municipality. Thus, purposive sampling technique was used to recruit only family caregivers of cerebrovascular accident survivors. This was because we believed they are the only ones with the requisite experiences necessary for this study. In addition, snowball technique was used to locate and recruit other family caregivers living within the Municipality. So, caregivers who’s contact details we got from the center led us to other caregivers who in turn directed us to others [41–43].

### Development of interview guide

Guided by literature [15, 19, 21], we developed a four-item (with 14 prompts) interview guide named; “Instrument for Caregivers” (see appendix: Interview Guide) for this study. The guide covered background information (age, gender, level of formal education, and occupation) of the caregivers, knowledge about cerebrovascular-risk factors, challenges faced as family caregivers during the caregiving, impacts of caregiver role on the caregiver, and coping strategies of the caregiver. Some items on the guide included: *“What do you know about* cerebrovascular accident*-risk factors?”, “How prepared were you for this role?”, “What challenges do you face as a caregiver?”, “How has this role affected you personally?”, “What do you do to cope with the challenges?”*

To ensure validity, the instrument was reviewed by two cardiologists, a cardio nurse, and a Senior Lecturer who is a specialist in qualitative research methods and health promotion [35, 37, 39]. Furthermore, the instrument was pre-tested using three family caregivers of cerebrovascular accident survivors [34, 36, 38] at Cape Coast. Through a contact at the Central Regional Hospital, Cape Coast, these three family caregivers of cerebrovascular accident survivors were contacted and interviewed at their homes. We audio recorded the interviews. Leveraging on the descriptive phenomenological qualitative research design, audio recordings were transcribed verbatim and field notes reorganised into meaningful text to reflect the objectives of the study [35, 38, 40]. After that, audio recordings were played over and over, while we thoroughly examined the transcribed data to ensure consistency and accuracy of the data. Thereafter, the transcribed data was compared and reconciled with the field notes to form a single text [35, 38, 40]. The family caregivers were contacted again to ensure that we capture and reported their experiences accurately. This verification helped use to make corrections to our data. Then, thematic analysis was carried out and a full report developed based on the data [35, 38, 40].

### Data collection procedures

Family caregivers of cerebrovascular accident survivors were contacted directly at their homes where they were briefed on the nature and purpose of the study. We sought their consent to participate in the study before they were interviewed. One-on-one interviews were conducted with each family caregiver which were audio-recorded. We also took written noted during the interviews. A language expert from the University of Cape Coast helped in translating the instrument from English to the Ewe language, which was again translated back to English language to ensure consistency in interpretation. Each interview lasted between 40 and 60 min [37, 38]. To deal with the psycho-emotional needs of the caregivers during the interview, the services of a psychologist was procured who accompanied the team during interviews.

### Ethical considerations

Ethical clearance for the study was granted by Institutional Review Board (IRB), Research Department at the Ho Teaching Hospital (Ref No: HTHRD/HTH/2023/10). Before we commenced the interviews, informed consent was obtained from all participants and/or their legal guardian(s). We informed and explained to the participants that they could opt out of the study at any stage without a consequence. We also sought both written and oral consent from all participants [38–40] (see appendix: Interview Guide). Additionally, caregivers were assured of anonymity and confidentiality of their information [35, 36]. Where it was observed that the family caregivers was exhausted, busy, or could not concentrate during the interview, the interview session was suspended and rescheduled to another convenient day.

### Data analysis

Consistent with the techniques of DPA [35, 38, 40], data collection and analysis were triggered simultaneously, by all authors. First, audio files were transcribed verbatim and the field notes edited by all authors. Names and other features that may identify the participants were anonymised. Secondly, the transcribed text and field notes were reconciled into one text, and analysed using the four steps in DPA [37, 39], including bracketing, horizonalisation, clustering, and textualisation.

At bracketing, the transcript was read and audio tapes listened to repeatedly devoid of researcher biases and preconceptions [34, 38, 40]; by six authors (1, 2, 3, 4, 5, & 6). At horizontalisation, the transcript was read and audio tapes listened to again and again. This was supposed to give equal importance to all statements and expressions to be included in the data [34, 36]; done by six authors (2, 3, 4, 5, 6, & 7). At clustering, the transcript was read and audio tapes listened to repeatedly, significant statements and expressions were identified and categorised into codes and further into themes; done by seven authors (1, 2, 3, 4, 5, 6, & 7). To attain credibility, some participants were then contacted to confirm the genuineness of the views expressed and captured in our data; done by three authors (1, 2, & 8). Overall, trustworthiness was upheld by observing authenticity, credibility, confirmability, dependability, and transferability [35, 36, 39]. Finally, at textualisation, the phenomenon and their essences were comprehensively written using the emerging themes, based on the purpose and objectives of the study [36, 40]; done by five authors (2, 3, 4, 5, & 8).

### Rigor of study

To ensure qualitative rigour, the following principles were observed: credibility, dependability, transferability, confirmability, and reflexivity [34, 38, 40]. Credibility was established through persistent observation of the family caregivers for non-verbal cues and extensively examining the data, to identify similarities within the text [34, 38, 40]. Dependability was attained by recording and providing a detailed account of the research process to allow for replication by other researchers [38, 40]. Furthermore, member-checking and peer-debriefing of findings were done. Confirmability was attained through researcher-bracketing to alleviate researcher biases [34, 40]. Transferability was ensured through a detailed representation of the experiences of the family caregivers and full disclosure of the entire research process [34, 38]. Finally, reflexivity was established by being critical of the previous views and knowledge we may hold about the family caregivers so that such views do not interfere with our experiences during the current study [38, 40].

## Results

The study produced four themes and ten sub-themes. First theme, ill-prepared caregiver roles: sub-theme, inadequate knowledge about cerebrovascular-risk factors and inadequate education about caregiver roles. Second theme, barriers confronted by caregivers: sub-themes, drain on financial resources, lack of cooperation from patents and workload, and poor service by hospital staff. Third theme, the cost of caregiver roles on carers: sub-themes, poor health, loss of job and income, and inability to further education and breakdown in relationships. Fourth theme, inadequate coping strategies and poor knowledge: sub-themes, inadequate knowledge about coping strategies and poor coping strategies.

### Theme one –ill-prepared for caregiving roles

This theme yielded two sub-themes, including inadequate knowledge about cerebrovascular accident-risk factors and inadequate education on caregiver roles.

**Sub-theme One Inadequate knowledge about cerebrovascular accident-risk factors**: Many of the caregivers identified poor dieting, inadequate exercise, alcohol abuse, smoking, stress, and age as cerebrovascular accident-risk factors. A female participant (40FHNDT15) explained: *“…poor diet, inadequate exercise, smoking, drinking alcohol, genetics, and psychological problems. Yes, that is it. But, men must be careful because they are the ones at most risk.”* (3yrs as caregiver).

Meanwhile, some of the caregivers also identified spirituality and sex as risk factors. Participant ‘40FHNDT15’ again: *“…Older men around 45yrs and above who engage in too much sex can have this condition. Too much sex is dangerous for older men…. laughter”*.

Participant ‘27MPSU26’ added: *“…But, it could also be spiritually engineered through curses. So, one could be attacked with* cerebrovascular accident *if not spiritually protected….”*

**Sub-theme Two – Inadequate education about caregiver roles**: We also found that caregivers were not adequately prepared for their roles. A male participant (32MDMMV12) noted: *“…I was not educated on how to take care of him (a survivor). Though I was not around on the day of the incident, I am the one who takes him to the hospital for review….”* (Over 2yrs as caregiver).

A female participant (38FJHMMV27) explained: *“Yes, they (heath staff) told me to use fish and more vegetable for his (survivor’s) meals. I was also encouraged to give him more fruits and bring him to the hospital for physiotherapy. But, my main source of guidance came from a local herbalist with history of managing such conditions (survivors)….”* (3yrs as caregiver). Another female participant (45FPMMV18) submitted: *“…No education about how to take care of him (the survivor), but given my mood on the day of the incident, I would not have paid attention even if they (health staff) did. Seeing him motionless on the hospital bed was shocking to me and seems hopeless…it is God who heals.”* (2yrs as caregiver).

### Theme two – barriers confronted by caregivers

This theme produced three sub-themes, including drain on financial resources, lack of cooperation from the patients and workload, and poor service by the hospital staff.

**Sub-theme One – Drain on financial resources**: We identified financial challenges as a major drawback to the role of the caregivers. A female participant (44FDTr6) lamented: *“…Lack of money to buy all the prescribed drugs, cost of taking him to the hospital for review, and the high cost of food items are unbearable. Everything is expensive and yet there is no support from anywhere (for the survivor).”* (Over 1 year as caregiver).

Another female participant (56FSMMV30) submitted: *“…We contracted a herbalist who comes to massage her (the survivor) thrice weekly at a fee. If you consider the cost of drugs and special meals for her, the money they (siblings) give me for the month does not last beyond two weeks.”* (2yrs as caregiver).

#### Sub-theme two – lack of cooperation from survivors and workload

We found that cooperation of the survivors and workload affected the work of the caregivers.

A female participant (47FSHU1) cried out: *“…it is difficult getting him (the survivor’s) to take his drugs, eat, and exercise. I have to plead for hours just to bathe him and in everything else. There were days that he refused to take the drugs.”***(2yrs as caregiver).**

Another female participant (24FDSA33) explained: *“…I know this disease (*cerebrovascular accident*) affects the brain and therefore, the way they (survivors) think is different from ‘normal’ people. I really struggle caring for him. In fact, he sometimes insults me for forcing him to take the drugs. I could not imagine my own father insulting me…”***(2yrs as caregiver).**

Participant ‘56FSMMV30’ again explained how time and labour intensive caregiving is: *“Taking care of her (survivor) involves so much work and time. On a typical morning starting from about 0800hrs, by the time I am done taking care of her, is already past 1200hrs. You have to lift and turn her many times….”*

Participant ‘24FDSA33’ again: *“…just as you are done taking care of him (the survivor) in the morning, you have to start preparing for lunch. This is a full time job and without that commitment he would die….”* she cried.

#### Sub-theme three – poor service by hospital staff

We found that poor staff attitudes and communication and long waiting time at the hospital frustrate caregivers.

Participant ‘44FDTr6’ complained: *“…you have to wait for hours before they attend to you and some of them are not polite in their communication. Sometimes I wonder if they were not trained to take care of cerebrovascular accident survivors….”*

Participant ‘45FPMMV18’ again: *“you have to wait for so long before they attend to us and I always buy the drugs they prescribed. So, you see why some people prefer the herbalist?”*

### Theme three – the cost of caregiver role on carers

This theme produced three sub-themes, including poor health, loss of job and income, inability to further education and breakdown of relationship.

#### Sub-theme one – poor health

It emerged that stress and high blood pressure were common health complains by caregivers.

A female participant (35FDTr) explained: *“…I am always worried about when he (the survivor) would get better and this sets me thinking always. I have lost appetite for food and sometimes eat only lunch. I realised at a point that there was something wrong with me and when I checked at the hospital, it was confirmed that my blood pressure was high…the stress is too much.”* (2yrs as caregiver).

Another female participant (33FDU19) submitted: *“…I become so tired due to the heavy workload involved in his (CVA survivor) care. It is also so frustrating when you think about the opportunities you have missed….”* (2yrs as caregiver).

#### Sub-theme two – loss of job and income

Caregivers also reported loss of job and income since commencing care for cerebrovascular accident survivors.

A female participant (29FJHU20) lamented: *“I was teaching in a private secondary school but had to leave the job to take care of her (the survivor). As it stands, I have no job, no income, and I am not even sure when she would recover fully for me to find something to do.”* (Over 2yrs as caregiver).

Another female participant (37FSHSA14) cried: *“I was a shop attendant but had to stop because of his (the survivor’s) condition. I cannot work, go to church, or leave a normal life at this time.”* (Over 1 year as caregiver).

#### Sub-theme three – inability to further education and breakdown of relationship

Caregivers also reported their inability to go for further education and separation from their partners due to the caregiving role.

A female participant (31FPSU13) lamented: *“I was hoping to enter the nursing training college but had to wait until next year, hoping he (the survivor) gets better by then. I am trusting God for a ‘miracle’….”* (Over 2yrs as caregiver).

Another female participant (20FJHMMV9) explained: *“My husband and I have separated because I had left him to take care of my father (a survivor) for over a year now. But, the Lord is with me.”* (2yrs as caregiver).

### Theme four – inadequate coping strategies and poor knowledge

This theme yielded two sub-themes; inadequate knowledge about coping strategies and poor coping strategies of the caregivers.

#### Sub-theme one – inadequate knowledge about coping strategies

We found that caregivers were not very knowledgeable about positive coping strategies.

A male participant (35MDTr25) said: *“I had no prior education on how to deal with the challenges associated with taking care of him (the survivor) at all. It was all about how to mobilise resources and make sure he gets better in no time….”* (Over 1 year as caregiver). Another male participant (21MHNDSA2) responded: *“…no please! I was not advised on what and what not to avoid. Friends suggested I remain very prayerful….”* (Over 2yrs as caregiver).

#### Sub-theme two – poor coping strategies

Caregivers reported fasting and prayers and alcohol use as some coping strategies.

A female participant (36FHNDU8) submitted: *“……visits and encouragements from my siblings, friends, and church members sustained me. I also became more prayerful than before.”* (2yrs as caregiver).

Another female participant (27FDT10) responded: *“…I took to fasting and prayers, since this was the second incident of this sickness (*cerebrovascular accident*) in this family. Hm!!! But, there were days I become depressed that I had to drink alcohol before I could eat. ”* (Over 1 year as caregiver).

## Discussion

We explored the experiences of family caregivers providing support for cerebrovascular accident survivors. Our findings revealed that though the caregivers seem to low level of knowledge of the condition, they mentioned poor diet, inadequate exercise, alcohol abuse, smoking, stress, and ageing as the main cerebrovascular accident-risk factors. Unfortunately, many of the caregivers were not adequately prepared for the caregiving roles. Besides, many reported financial challenges, lack of cooperation from the patients and workload, poor communication and attitude of hospital staff, and long waiting time at the hospital during medical reviews. The caregivers also reported stress and high blood pressure, loss of job and income, inability to further their education and separation from their partners/family disintegration as they care for the cerebrovascular accident survivors. Additionally, these caregivers did not report adequately knowledge of coping strategies, some reported fasting and prayers, and use of alcohol as ways to cope with the roles of caregiving.

We found that though the caregivers were familiar with some very important cerebrovascular accident-risk factors, their knowledge was limited. Moreover, the misconceptions that spirituality and sexual activity determine cerebrovascular accident was popular among these caregivers. This strongly affirms previous studies [8, 10–13, 21] which reported of narrow knowledge about cerebrovascular accident-risk factors among patients, caregivers, and the general population. Furthermore, the current study upholds findings from previous writers like Beatrice et al. [44], Doumit et al. [45], and Siler et al. [46], that caregivers normally attribute chronic diseases (like cerebrovascular accident) to spirituality. Additionally, we observed that the caregivers had no proper orientation or training from the healthcare staff on how to effectively manage the cerebrovascular accident survivors, which may explain their limited knowledge.

Given that these caregivers are Ghanaians with strong attachment to culture and family, local herbalists and other family members [24, 25] are very likely sources of guidance to their roles as caregivers for cerebrovascular accident survivors. This could strongly influence the quality of care provided by these family caregivers to the patients. This contradicts what pertains in most Western countries [8, 9] where family caregivers relied on medical professionals for guidance in caring for such patients. However, findings of the current study firmly coheres with other researchers [10, 11, 21, 22] who suggested that family caregivers were ill-prepared for their caregiving roles. The implications are that cerebrovascular accident survivors may take longer time to recover, while caregivers experience poor health quality of life [8, 10, 21]. Such unpreparedness may not only be a threat to the patient, it could compound the already dire psycho-emotional ill health of the caregiver. The other effects are compromised health and poor quality of life, which may deter many persons from taking up such caregiving role.

We further found that the effectiveness of the support provided by caregivers was undermined by inadequate money, lack of cooperation from the cerebrovascular accident survivors, poor attitudes of healthcare staff and poor communication, and long waiting time at the hospitals. For instance, caregivers had to buy most of the prescribed drugs from private pharmacies which they are not always able to afford because such medications are very expensive. Many previous studies [10, 11, 21, 22] also reported of the expensive nature of the drugs or treatment of chronic diseases, which makes it difficult for patients and their caregivers to follow treatment regimen properly. This could compound the caregiving role, and undermine the health outcome and well-being of the caregivers, making them potential candidates for cerebrovascular accident [9].

The role of caregiving can greatly compromise the health, well-being and the general quality of life of the caregivers. For instance, evidence found a relation between caregiver role and incidence of poor mental health and high blood pressure [9, 10]. Furthermore, there is a high reported incidence of job and income losses due to the caregiving roles [9, 47, 48]. Similarly, the current study found self-reported incidence of stress, high blood pressure, loss of job and income, inability for further education, and breakdown of relationships because of the caregiving roles taken by these individuals. Unfortunately, these socio-economic and psycho-emotional challenges are strong triggers of cerebrovascular accidents [9, 10, 47, 48] which can happen to these caregivers. Therefore, these caregivers would need social, economic and psychological support to deflate the adverse effects of their caregiving roles, to promote their health and quality of life.

We also found that these caregivers do not have enough knowledge of the coping strategies needed as they care for the cerebrovascular accident survivors. The subject of coping strategies is hardly discussed in contemporary literature in terms of out-of-facility care for chronic disease patients, since the focus is more on the patients rather than the caregivers. Other previous studies also reported poor knowledge of coping strategies among caregivers [23, 49, 50]. This may not only lead to poor care for the patients and complicate patient’s health outcomes, but could also lead to high level of psychological challenges to the caregivers. This may further result in maladaptive behaviours like engaging in drug use. For example, Qiu et al. [23] reported alcohol abuse as a common coping strategy adopted by some caregivers. Similarly, the current study observed fasting and prayers, and alcohol abuse as coping strategies adopted by some of the caregivers. While these observation could be considered as unique to caregivers from Ghana or from developing countries, they contrast what pertains from the Western world [8, 9]. Unfortunately, these coping strategies may predispose the caregivers to cerebrovascular accident-risk factors like hypertension, stress, depression, alcohol dependency, and poor dieting [10, 11, 21], that lead to cerebrovascular accident.

The findings from the current study are inconsistent with the letter and spirit of the Sustainable Development Goals 3.4. Sustainable Development Goal 3.4 aims at reducing 75% in premature deaths due to non-communicable diseases (including cerebrovascular accidents) by 2030 [5, 7]. Unfortunately, there seems to be no policy that persuasively defines the roles of caregivers and what social support systems that exist and best fit their circumstances. We are of the view that with less than five years to 2030, something pragmatic and urgent needs to be done if Sustainable Development Goals 3.4 is to be achieved in Ghana.

## Limitations

This study has three main limitations. Firstly, the qualitative design employed and sample size of 37 used limited the generalisability of our findings because that may not be true representation of caregivers in Ghana and other developing countries. Secondly, some caregivers may have suffered recall bias which limit the reliability of their information and our findings. Thirdly, some caregivers were emotional in their presentations and this could influence the quality of information they provided.

## Conclusion

We report that caregivers had limited knowledge about cerebrovascular accident-risk factors and were ill-prepared for their caregiving roles. Furthermore, we found that the caregiving roles are affected largely by inadequate funds, lack of cooperation from the patients, poor attitude from the healthcare staff, including poor communication, and the high cost of drugs. Moreover, caregivers were impacted in various ways, including high levels of stress, high blood pressure, loss of job and income, inability to further education, and breakdown of family relationships. We also found limited knowledge about coping strategies among the caregivers, who further reported of fasting and praying to God, and alcohol use and abuse as some of their coping strategies. Thus, many of these caregivers adopted ill-coping strategies that could compromise their health, including developing cerebrovascular accidents. Therefore, there is the need for healthcare providers at Ho Teaching Hospital Cardio Centre to provide training for these family caregivers as they commenced care for the cerebrovascular accident survivors.

Our findings cast doubt on the possibility of achieving Sustainable Development Goals 3.4 in Ghana and many other developing countries. This is because, there seems to be no deliberate action, including policy that target caregivers and provide social support systems that best fit their circumstances. Our study is arguably the first one to have exclusively explored the lived experiences of family caregivers of cerebrovascular accident survivors in Ghana, and Africa. Therefore, we recommend that Ghana develops a policy on family caregiving, the Ho Teaching Hospital Cardio Centre organises series of screening exercises for caregivers of cerebrovascular accident survivors. Furthermore, there is the need to survey family caregivers of the prevalence of risk of cerebrovascular accident-risks.

### Electronic supplementary material

Below is the link to the electronic supplementary material.

Supplementary Material 1

Supplementary Material 2. Table 1: Biographic characteristics of caregivers. Interview Guide: Instrument for Caregivers

## Data Availability

Not applicable, as all data underpinning the study are fully captured in the manuscript.
