# Supplementary material for: Care for the cerebrovascular accident survivors: experiences of family caregivers
Source: BMC Palliat Care. 2024 Jun 1;23:138. doi: 10.1186/s12904-024-01468-6 (PMC11143680; doi:10.1186/s12904-024-01468-6)
Supplement: Supplementary file 1 — Supplementary Material 1 [file 12904_2024_1468_MOESM1_ESM.docx]

**INSTRUMENT FOR CAREGIVES**

**PARTICIPANT INFORMATION**

**Dear Participant,**

We researchers from the College of Education, Amedzofe and University of Cape Coast conducting a research on the topic “**Care for the Cerebrovascular Accident (CVA) Survivors: Experiences of Family Caregivers**”. This study examines experiences of family caregivers providing care for CVA survivors. We are kindly requesting your participation in this study because you have experience caring for CVA survivor which we consider very useful for this study. We kindly solicit your honest and sincere submissions during the interview with you, which will take about 45 minutes of your time. We want to inform you that you have the liberty to withdraw from the study, even after you have consented to participate.

This study poses no serious harm to you excerpt that we envisage some psychological and emotional disturbances that may come by recalling your experiences with caring for the CVA survivor. Because of that we have a psychologist who will always be available during the interview. Moreover, we assure you that all your information will be treated with outmost confidentiality and anonymity it deserves. Moreover, be informed that the findings and conclusions from this research may be presented at workshops, conferences and or published in journals.

If you agree to take part in this research, please sign in the space provided below.

Signature: …………………………..

Date: …………………………………….

For further information, please contact me (Fortune) on +233 243901506 or my fellow investigators Edward on +233 247703379 and Nkosi Nkosi on +233 242766087.

Thank you and I appreciate your time.

**INTERVIEW GUIDE**

Demographic characteristics of the participants:

Age: ………………

Sex: ………………

Level of formal education: ……………………

Occupation: …………………..

Years of experience as family caregiver: ………………..

**A. Awareness and knowledge about CVA-risk factors and caregiver role**

1. What do you know about CVA-risk factors and how prepared were you as a caregiver?

**Awareness**

i. Source of information about CVA-risk factors,

ii. Education about CVA-risks factors and family caregiver role.

**Knowledge**

i. Modifiable and non-modifiable CVA-risk factors,

ii. Medications for CVA survivor,

iii. Feeding of CVA survivor,

iv. Personal feeding habits and activity level,

v. Personal health concerns.

**B. Challenges involved in the caregiver role**

2. What challenges do you face as a caregiver?

i. Financial challenges,

ii. Lack of cooperation from CVA survivors and workload,

iii. Hospital-based challenges.

**C. Impact of caregiver role on carers**

3. How has this role affected you?

i. Loss of job and income,

ii. Inability to further education and breakdown of relationship.

**D. Coping strategies adopted by caregivers**

4. What do you do to cope with the challenges?

i. Knowledge about coping strategies,

ii. Coping strategies adopted.
