# Supplementary material for: Care for the cerebrovascular accident survivors: experiences of family caregivers
Source: BMC Palliat Care. 2024 Jun 1;23:138. doi: 10.1186/s12904-024-01468-6 (PMC11143680; doi:10.1186/s12904-024-01468-6)
Supplement: Supplementary file 2 — Supplementary Material 2. Table 1: Biographic characteristics of caregivers. Interview Guide: Instrument for Caregivers [file 12904_2024_1468_MOESM2_ESM.docx]

**Appendix**

**Table 1: Biographic characteristics of caregivers**

| **No.** | **Gender** | **Age** | **Caregiver**  **Experience** | **Level of**  **education** | **Occupation** | **Relationship with survivor** | **Pseudonym** |
| --- | --- | --- | --- | --- | --- | --- | --- |
| 1. | Female | 47 | 2yrs | Senior High | Unemployed | Sister | 47FSHU1 |
| 2. | Male | 21 | Over 2yrs | Higher National Diploma | Shop attendant | Son | 21MHNDSA2 |
| 3. | Female | 28 | 3yrs | Bachelor’s Degree | Teacher | Daughter | 28FDT3 |
| 4. | Female | 45 | 2yrs | Junior High | Trader | Wife | 45FJHTr4 |
| 5. | Female | 35 | Over 2yrs | Bachelor’s Degree | Trader | Wife | 35FDTr5 |
| 6. | Female | 44 | Over 1yr | Bachelor’s Degree | Trader | Wife | 44FDTr6 |
| 7. | Female | 23 | Over 1yrs | Senior High | Trader | Daughter | 23FSHTr7 |
| 8. | Female | 36 | Over 2yrs | Higher National Diploma | Unemployed | Daughter | 36FHNDU8 |
| 9. | Female | 20 | 2yrs | Junior High | Mobile money vendor | Daughter | 20FJHMMV9 |
| 10. | Female | 27 | Over 1yr | Bachelor’s Degree | Teacher | Daughter | 27FDT10 |
| 11. | Male | 53 | 2yrs | Diploma | Teacher | Husband | 53MDiT11 |
| 12. | Male | 32 | Over 2yrs | Bachelor’s Degree | Mobile money vendor | Daughter | 32MDMMV12 |
| 13. | Female | 31 | Over 2yrs | Primary School | Unemployed | Daughter | 31FPSU13 |
| 14. | Female | 37 | Over 1yr | Senior High | Shop attendant | Daughter | 37FSHSA14 |
| 15. | Female | 40 | 3yrs | Higher National Diploma | Teacher | Daughter | 40FHNDT15 |
| 16. | Male | 46 | 2yrs | Bachelor’s Degree | Trader | Brother | 46MDTr16 |
| 17. | Female | 27 | 2yrs | Junior High | Trader | Daughter | 27FJHTr17 |
| 18. | Female | 45 | 2yrs | Primary | Mobile money vendor | Daughter | 45FPMMV18 |
| 19. | Female | 33 | Over 2yrs | Bachelor’s Degree | Unemployed | Daughter | 33FDU19 |
| 20. | Female | 29 | Over 2yrs | Junior High | Unemployed | Daughter | 29FJHU20 |
| 21. | Female | 48 | 2yrs | Higher National Diploma | Unemployed | Wife | 48FHNDU21 |
| 22. | Female | 31 | Over 1yr | Junior High | Mobile money vendor | Daughter | 31FJHMMV22 |
| 23. | Female | 42 | Over 2yrs | Bachelor’s Degree | Mobile money vendor | Sister | 42FDMMV23 |
| 24. | Female | 50 | Over 2yrs | Diploma | Trader | Wife | 50FDiTr24 |
| 25. | Male | 35 | Over 1yr | Bachelor’s Degree | Trader | Son | 35MDTr25 |
| 26. | Male | 27 | 2yrs | Primary School | Unemployed | Son | 27MPSU26 |
| 27. | Female | 38 | 3yrs | Junior High | Mobile money vendor | Wife | 38FJHMMV27 |
| 28. | Female | 44 | Over 1yr | None | Teacher | Wife | 44FT28 |
| 29. | Male | 23 | Over 2yrs | Bachelor’s Degree | Trader | Son | 23MDTr29 |
| 30. | Female | 56 | 2yrs | Senior High | Mobile money vendor | Sister | 56FSMMV30 |
| 31. | Female | 63 | Over 1yr | None | Unemployed | Wife | 63FU31 |
| 32. | Female | 42 | Over 3yrs | Junior High | Shop attendant | Daughter | 42FJHSA32 |
| 33. | Female | 24 | 2yrs | Bachelor’s Degree | Shop attendant | Daughter | 24FDSA33 |
| 34. | Female | 23 | Over 1yr | Bachelor’s Degree | Shop attendant | Daughter | 23FDSA34 |
| 35. | Male | 38 | Over 1yr | Bachelor’s Degree | Unemployed | Son | 38MDU35 |
| 36. | Female | 36 | 2yrs | Primary School | Mobile money vendor | Daughter | 36FPMMV36 |
| 37. | Female | 31 | Over 1yr | Bachelor’s Degree | Mobile money vendor | Daughter | 31FDMMV37 |
